# Ingestible Sensors and Medication Adherence: Focus on Use in Serious Mental Illness

**DOI:** 10.3390/pharmacy8020103

**Published:** 2020-06-16

**Authors:** Azita Alipour, Stephen Gabrielson, Puja Baldev Patel

**Affiliations:** 1 College of Pharmacy, Marshall B. Ketchum University, Fullerton, CA 92831, USA; aalipour@ketchum.edu; 2 Health Sciences Library System, University of Pittsburgh, Pittsburgh, PA 15261, USA; sgabrielson@pitt.edu

**Keywords:** adherence, antipsychotic, ingestible sensor, schizophrenia, serious mental illness

## Abstract

Background: Poor medication adherence is a major public health concern. Patients living with a serious mental illness (SMI) commonly present with non-adherence to their medication regimen, which can lead to relapse and hospitalizations. The high rates of antipsychotic non-adherence continue to persist despite several interventions and medication advances. This review evaluates the possible role of the ingestible sensor technology for medication adherence in different conditions, with a focus on use in the SMI schizophrenia. Methods: Literature searches were conducted in July 2019 in the PubMed database. Results: In small studies of ingestible sensor use, the average adherence ranged from 73.9% to 88.6% for SMI and ≥ 80% for cardiac and transplant (99.4%) patients. In SMI studies, patients were clinically stable, and the majority had a clinical global impression severity of “mild disease”. Patients generally experienced relatively minor dermatological adverse effects related to wearable sensor use. Conclusions: A medication with an ingestible sensor may help provide real-time objective medication-taking adherence information for clinicians. However, further studies are needed to understand the impact of use on adherence and improvement on treatment outcomes with the ingestible sensor technology.

## 1. Introduction

Lack of adherence to medications is a significant public health issue. According to the World Health Organization (WHO), “adherence to medication can be defined as the degree to which use of medication by the patient corresponds with the prescribed regimen” [1]. Although there is a lack of general consensus, research indicates that medication adherence may be quantified as using the medication as directed 75 to 80% of the time [2,3]. The average rate of adherence to long-term therapy for chronic illnesses is approximately 50% in developed countries [1]. Depending on the publication, individuals living with severe mental illness may have non-adherence rates that are equivalent or worse than non-psychiatric chronic illnesses, with reported antipsychotic non-adherence rates of up to 89% [4,5,6].

Poor medication adherence can result in negative health consequences, decreased quality of life, and increased healthcare costs [1,7]. Relapse and hospitalization are major consequences of non-adherence in individuals with schizophrenia [4]. The risk of hospitalization can double with as little as a few days of non-adherence [8]. The high rates of antipsychotic non-adherence continue to persist despite several interventions and medication advances [9].

Given the high rate of non-adherence and its health consequences, researchers have attempted to identify risk factors to help with developing strategies for improvement [2]. Risk factors typically have been grouped under “patient, environment, and medication-related factors” [2]. Several studies and reviews have looked to identify risk factors in individuals with schizophrenia with inconsistent results [9]. Czobor et al. combined results from the Clinical Antipsychotic Trials Intervention Effectiveness (CATIE) study and the European First Episode Schizophrenia Trial (EUFEST) to assess factors affecting adherence in schizophrenia. They found decreased adherence correlated with impaired insight, use of substances, and increased hostility [10]. A systematic review found improved adherence consistently connected with the following factors: disease insight and positive attitude towards medications [2]. The authors found inconsistent results for factors related to sociodemographic characteristics, medication adverse effects, and severity of symptoms [2]. Overall, there is consensus amongst most reviewers on some factors that non-adherent individuals with schizophrenia may have, such as increased severity of symptoms, disorganization, incidence of positive symptoms, and abuse of substances [9]. Additionally, “most reviewers conclude that adherence is better if patients have insight, positive attitudes toward medication, and belief or experience that medication is effective” [9]. Lack of a family support system, living alone, and unsupervised medication taking can have negative effects on adherence [9]. In contrast, a good therapeutic alliance may be beneficial for adherence [9].

Antipsychotics are a vital part of treatment for individuals with schizophrenia [11]. The first long-acting antipsychotics (LAIs) were developed in the 1960s to enhance adherence [12]. LAIs aim to decrease relapse, exacerbation of symptoms, and hospitalizations through improved adherence [13]. LAIs tend to have improved adherence profiles compared with oral antipsychotics [11]. Non-adherence estimates for LAIs vary from 9 to 52% [11]. Currently, there are two first-generation (haloperidol, fluphenazine) and four second-generation antipsychotics (aripiprazole, olanzapine, paliperadone, risperidone) available as various commercially approved LAIs [12,14,15]. LAIs each have different dosing schedules, with Risperdal Consta© as early as every two weeks and Invega Trinza© given every three months [16,17]. According to Greene et al., barriers to use of LAIs include “…negative attitudes from clinicians and patients, logistical/transportation challenges, a lack of insurance coverage, and general-purpose clinics serving this population that are not properly equipped to administer injections” [11].

Non-pharmacological approaches for improving adherence have included cognitive behavioral therapy, psychoeducation, motivational, family, and technological approaches [18]. Advances in information technology, artificial intelligence, ease of access to smartphones, apps, and devices with capabilities of recording and transmitting information have allowed for advances in adherence technology interventions [19]. Examples include medication containers with electronic monitoring, wearables, and electronic ingestible event marker [20,21]. The Medication Events Monitoring System (MEMS) is an electronic monitoring pill bottle with a chip in the cap recording the number of times each day the bottle is opened [18]. A limitation of the MEMS is based on if the bottle is opened and does not account for ingestion. In contrast, an ingestible sensor can provide direct medication taking behavior feedback [22]. Proteus Digital Health has created a digital medicine offering (DMO) consisting of four components: an ingestible sensor, an adhesive sensor patch worn by the patient, a mobile app for patients, and a web portal for providers [22]. 

Aripiprazole is a second-generation antipsychotic approved in adults for the treatment of schizophrenia, bipolar disorder, and depression [23]. Aripiprazole is available as an oral tablet, oral solution, orally disintegrating tablet (ODT), LAIs (Abilify Maintana and Abilify Aristada), and a drug–device combination (Abilify Mycite) [23]. Abilify Mycite is an oral tablet with an ingestible sensor, which can track patient adherence [23,24]. Abilify Mycite was approved in the United States in November of 2017 as “a drug-device combination product” [24,25]. Abilify Mycite is the first medicine to be approved by the Food and Drug Administration that allows for ingestions to be digitally tracked and utilizes the Proteus Digital Health System [24,25,26].

In this article, we discuss studies looking at medication adherence with the ingestible sensor technology. This article will include conditions where this technology has been studied, with a focus on the implications for adherence in patients living with the serious mental illness (SMI) schizophrenia.

## 2. Materials and Methods

Searches of the literature were conducted in PubMed in July 2019. The first search was on medication adherence technologies for all conditions. Keywords used in this search included “medication adherence”, “biosensors”, “ingestible sensors”, “smart pills”, “digital medicine”, and “digital pill system”. Medical Subject Headings (MeSH) were also used, and included “medication adherence”, “technology, pharmaceutical/instrumentation”, and “biosensing techniques”.

The second search was broadened to include all types of medication adherence techniques with a focus on schizophrenia. A combination of keywords and MeSH terms were used and included “medication adherence” and “schizophrenia”. The results in this second search were limited to articles published within the past five years.

## 3. Results

### 3.1. Ingestible Sensors

#### 3.1.1. General Overview

A digital medicine system (DMS) provides an objective real-time recognition of non-adherence with medications [27,28]. The Proteus DMO can capture medication ingestions (if the sensor is combined with medications) and other health information, which are later viewable by providers and patients [22]. The ingestible sensor is composed of materials found in the food supply and naturally eliminated through the body by the fecal route [27]. The ingestible sensor is activated after being swallowed once it interacts with stomach fluid [22,27]. Upon activation, the ingestible sensor generates a signal identified by the wearable adhesive patch, which also collects other health information [22,27]. Patch-collected data are then sent to a mobile device and subsequently the cloud [22]. This data are then viewable by patients and providers on the mobile app and web portal, respectively [22].

An ingestible device, open-label pilot study conducted over a four week period with 20 healthy volunteers demonstrated no adverse events or discontinuations associated with device use. The device demonstrated close to 98% accuracy in detecting ingestion [29]. There was a high level of documented adherence, although the authors postulate adherence could have been due to other factors including the Hawthorne effect, study visits, and daily text message reminders [29]. The pilot study per the authors demonstrated the feasibility of using the technology’s real-time adherence information [29]. Although the FDA recently approved the first ingestible sensor with aripiprazole in psychiatry patients, the ingestible sensor technology has been studied in multiple other conditions, including cardiac disease states and infectious disease prevention and treatment [29,30,31,32,33]. It has also been used in transplant patients and those requiring opioids [34,35,36].

Holender et al. conducted a qualitative study utilizing focus groups in elderly patients on cardiovascular medications to evaluate the attitudes and practicality of the utilization of different technology modalities to improve medication adherence in the elderly [31]. Ingestible sensors were viewed as “helpful” for community-dwelling elderly patients with cognitive impairment due to better monitoring of adherence by providers and caregivers [31].

#### 3.1.2. Cardiovascular

Frias et al. conducted an open-label, prospective, cluster-randomized pilot trial to assess the effectiveness of a digital medicine offering to improve outcomes in patients with uncontrolled type 2 diabetes and hypertension. Subjects were randomized to receive usual care or the digital medicine offering (DMO) for either 4 weeks or 12 weeks [22]. The DMO consisted of the co-encapsulated medication and ingestible sensor, adhesive patch sensor that was to be worn by the patient, a mobile app, and web portal for providers [22]. The primary aim was to evaluate the DMO effect on blood pressure [22]. Other aims included evaluating the effect on lipid control, glycemic control, decision-making by the provider, and engagement [22]. Blood pressure readings were collected at every visit [22]. Laboratory values for low-density lipoprotein cholesterol (LDL-C), total cholesterol, and fasting plasma glucose were drawn at screening, week 4, and week 12 [22]. Hemoglobin A1c (HbA1c) was drawn both at screening and week 12 [22]. Subjects participated in a Patient Activation Measure (PAM) during the study as well [22]. It was found that compared with usual care, DMO produced a statistically larger decrease in systolic blood pressure (SBP) at week 4 [22]. The larger decreases in SBP observed in week 4 were maintained in the DMO at the week 12 evaluation [22]. Subjects randomized to DMO also had larger decreases in diastolic blood pressure, LDL-C, and HbA1c [22]. Additionally, a larger percentage of subjects in the DMO arms were at the blood pressure goal at the week 4 and 12 evaluations [22]. Compared with usual care, the DMO arms had more interventions. Adherence to medication in the DMO arms was reported to be greater than or equal to 80% during the study [22].

DiCarlo et al. incorporated the Proteus ingestible sensor into valsartan in 37 subjects with essential hypertension and passively collected data including medication ingestion, daily steps, weight, and blood pressure [37]. The positive detection accuracy of doses given in the clinic was 98% [37]. The mean taking adherence was reported at 90%, and the mean timing adherence was reported at 83% [37]. Swallowing the capsule of digital medicine was not minded by 90% of subjects [37]. The system was also evaluated for overall positive experience and reported this at 75% [37].

Noble et al. described the use of a digital health feedback system (DHFS) by pharmacists to develop recommendations for managing blood pressure [38]. The usability of the DHFS was assessed by pharmacists via patient interviews [38]. Satisfaction surveys were also mailed to subjects and pharmacists [38]. Survey and interview questions inquired about “comfort, ease of use, usefulness, and satisfaction with the pill, patch, and mobile displays” [38]. This study found that following two weeks of DHFS use, blood pressure was reduced [38]. Pharmacist recommendations following the use of a DHFS included either improving adherence to medication, “increasing activity level”, or both [38]. Surveys were completed by 11 of 13 pharmacists “and found that the DHFS helped to create a collaborative experience with their patients” [38]. Of the pharmacists that completed the survey, 91% found that DHFS data assisted them in making recommendations and counseling patients [38]. Subjects thought the experience was helpful and positive [38].

#### 3.1.3. Infectious Diseases

Subbaraman et al. reported that ingestible sensors have been investigated in pilot studies for patients with tuberculosis [33]. Garrison and Haberer report ingestible sensors are being researched in the setting of human immunodeficiency virus (HIV) preexposure prophylaxis and antiretroviral therapy, however, they report these trial data have yet to be published [32]. However, a bioequivalence study comparing unencapsulated tenofovir disoproxil fumarate/emtricitabine (TDF/FTC) to TDF/FTC co-encapsulated with the Proteus ingestible sensor is available [39]. This pharmacokinetic study demonstrated the bioequivalence of the co-encapsulated medication [39].

#### 3.1.4. Pain Management

Chai et al. evaluated the use of an oxycodone digital pill system to monitor oxycodone use in opioid-naive subjects following emergency department discharge for pain from acute fractures [34]. There were 112 ingestions recorded by the system, however, the pill count at the end of the study revealed 134 ingestions [34]. The accuracy of the digital system was reported at 84% [34]. The median number of oxycodone digital pills ingested was 6 (3–9) [34]. In subjects that required repair with operation, the median number of ingestions was 8 (6–11) [34].

Chai also performed another study with a digital pill system utilizing oxycodone [35]. This study focused on patients that had pain following discharge for an acute extremity fracture [35]. The duration of this study was one week [35]. Recorded ingestions by the system were compared to pill counts [35]. Subjects also participated in interviews. The digital system captured 96 ingestion events, however, the pill count revealed 110 ingestions [35]. The accuracy of the digital system at detecting ingestion was reported at 87.3% [35]. “A positive experience integrating digital pill into medication regimen” was reported by 90% of subjects and 80% “would be willing to use the digital pill for adherence monitoring in chronic disease and would be willing to share ingestion data with physicians” [35].

#### 3.1.5. Transplant

The ingestible sensor system has also been evaluated in a pilot study of 20 adult renal transplant patients taking enteric-coated mycophenolate sodium [36]. Treatment was stopped early in eight patients due to either rash from the adhesive personal monitor, diarrhea from the enteric-coated mycophenolate sodium, or “insufficient system usability” [36]. Of the 34 ingestions that were directly observed, the detection accuracy of the ingestible event maker was 100% [36]. However, when ingestions were not directly observed, only 2824 ingestions occurred out of the 4136 ingestions prescribed. The discrepancy of ingestion was often related to patients not wearing the adhesive personal monitor [36]. The study reported a 99.4% taking adherence to the 2824 ingestions that were prescribed [36].

#### 3.1.6. Psychiatry

Exploratory clinical trials and human factor studies were completed to look at the safety, tolerability, and usability of a DMS in people living with a serious mental illness [40]. A dermal safety study was conducted in 30 individuals to assess the potential safety and tolerability of the sensor and assess for any skin irritation from the wearable sensor [40]. During the study, on average, subjects had the sensor on six days per week with “the period of wearability increased over the course of the trial” [40]. The wearable sensor was found during the trial to be well-tolerated, with a slight rise in pruritus, minimal to no irritation, and no adverse events [40].

Peters-Strickland et al. looked at the human factors (HF) of using a digital medicine system in psychiatry patients living with a serious mental illness to eliminate use-related risks which may impact usability. For example, including explicit instructions and eliminating ambiguous wording in the app allowed for decreasing the need for abstract thinking [41]. Other improvements allowed for decreasing the reliance on working memory and overall cognitive effort [41].

The usability of a DMS consisting of aripiprazole embedded with an ingestible sensor in subjects with schizophrenia stable on aripiprazole was accessed in a multicenter, open-label, phase IIa study [42]. After a screening phase, subjects entered the training phase, which consisted of three weekly site visits, followed by the independent phase lasting five weeks [42]. Of the 67 patients enrolled, 49 (73.1%) finished the study [42]. The majority (70.1%) of subjects had a clinical global impression severity scale of “mildly ill” [42]. Calls from subjects were received by the call center most frequently during the first week and by the end of the study (or by early termination), 55 of the subjects (82.1%) were able to by themselves or with little help replace the wearable sensor [42]. The average medication adherence was 73.9% in the study [42]. The majority (78%) of the subjects reported they were “somewhat satisfied/satisfied/extremely satisfied” with the DMS [42]. The authors concluded the findings support a potential clinical role for DMSs as a large number of subjects with schizophrenia were satisfied and able to use it [42].

Kane et al. conducted a feasibility and safety study of an ingestible sensor technology in a four-week observational study of 16 subjects with schizophrenia and 12 subjects with bipolar disorder stable on their current medication (mood-stabilizer or antipsychotic) [27]. Subjects with greater severity of depression, psychosis, or mania were excluded [27]. Adherence was a secondary endpoint of the study [27]. The average adherence rate was 74%, with 96% (27/28) of subjects completing the study [27]. There were no serious adverse events due to the ingestible sensor [27]. Per the study authors, the digital health feedback system (DHFS) was not the cause of any worsening psychosis in any of the subjects [27]. The majority of the study completers thought the DHFS was simple to understand, could be beneficial for them, and they would like telephonic missed dose message reminders [27].

Kopelowicz et al. conducted an open-label, multicenter pilot study of subjects on stable doses of aripiprazole for schizophrenia, major depressive disorder (MDD), or bipolar disorder who were amenable to the use of an aripiprazole DMS (aripiprazole with ingestible sensor) [28]. Of the 49 subjects who met criteria for study inclusion, 22 (45%) had bipolar disorder, 12 (24%) had MDD, and 15 (31%) had schizophrenia [28]. The subjects’ average adherence based on the ingestion data was high, around 88.6% (bipolar disorder 88.8%, MDD 91.9%, schizophrenia 85.6%) [28]. Greater than 50% of the call center calls were subjects with schizophrenia, which per the authors, indicates this group may need more help during the initiation of the DMS [28]. Treatment-emergent adverse events (TEAEs) occurred in 20 patients (40.8%) and device-associated TEAEs in 17 (34.7%) patients [28]. However, only one TEAE (erythema caused by patch) resulted in study drug discontinuation [28]. Rash was the most frequently occurring TEAE related to the device [28]. See Table 1 for a summary of adherence with a DMS in different conditions.

Hatch et al. conducted an expert consensus survey, which included 58 experts in psychiatry looking at different factors related to adherence, including “when and how to use the DMS in clinical practice once available” [43]. The survey described the DMS as “a digital medication platform that will give clinicians access to real-time empirical data concerning whether their patients are actually taking their oral antipsychotic medication” [43]. Measuring adherence was found by the expert panel to be especially important in the following situations: history of re-occurring poor adherence, transitions of care (ex: recent discharge from hospital), substance abuse history, and increase in side effects or symptoms in patients who had been stable [43]. They note that a DMS’s usefulness may depend on the underlying cause of non-adherence with the most appropriate indications viewed by the expert panel for patients with cognitive barriers, absence of daily routine, and for continuation of medication after discharge from the hospital [43]. A significant barrier to DMS use noted by the majority of the expert panel was patients who are unable to use technology [43]. Four potential uses for a DMS were identified by the expert panel, which included assessment of adherence, missed doses alerts to providers, interventions for adherence, and monitoring during routine follow-ups [43]. The experts recommended customizing alerts based on patient-specific factors and for providers waiting to receive missed dose alerts after two to three days and three days for bipolar disorder and schizophrenia, respectively [43].

## 4. Discussion

Patients living with an SMI commonly present with non-adherence to their medication regimen, which can lead to relapse and hospitalizations [28,44]. The high rate of antipsychotic non-adherence continues to persist despite several interventions and medication advances [9]. Limitations to adherence assessment can include a lack of real-time, accurate adherence information to identify and intervene when non-adherence starts [28]. Timing of identification can be significant as the risk of hospitalization may double with as little as a few days of medication non-adherence [8].

An ingestible sensor embedded in an antipsychotic may potentially help address this gap in care as it can provide a real-time medication adherence assessment [28,45]. An expert panel recommended the potential utility of a DMS by a health care provider receiving missed dose alerts within two–three days of missed doses (three days for schizophrenia) [43]. In turn, the health care provider could contact patients, family members, and the case manager when non-compliance with the DMS is identified [43]. Identifying and addressing poor medication compliance as soon as it occurs may help prevent relapse [43].

Although an advantage of an ingestible system is real-time antipsychotic ingestion information, improvement of antipsychotic adherence has not been proven with a DMS [28]. In small studies of ingestible sensor use, adherence ranged from 73.9% to 88.6% for SMI and ≥ 80% for cardiac and transplant (99.4%) patients [22,27,28,36]. Controlled trials with an active comparator looking at clinical outcomes are needed to understand the impact of adding an ingestible sensor for improving medication adherence in SMI [46]. However, the trial by Frias et al. did prospectively assess the effectiveness of a digital medicine offering (DMO) in improving outcomes in patients with uncontrolled hypertension and type 2 diabetes [22]. Compared with usual care, subjects in the DMO arms had larger decreases in blood pressure with a larger percentage of DMO subjects meeting the blood pressure goal at the week 4 and 12 evaluations [22]. The DMO subjects also had larger decreases in HbA1c and LDL-C. Adherence to medication in the DMO arms was reported to be greater than 80%, compared with generally an average adherence rate of 50% in chronic illnesses [1,22]. Per Frias et al., “improved clinical outcomes with the DMO were related in part to improved self-care (medication adherence and patient activation)” [22]. The DMO may have contributed to these improved outcomes by investigators using the DMO data to help with timely treatment interventions (example: adjustments to therapy, education/adherence counseling) [22]. Given poor medication adherence is not limited to psychiatric conditions, the outcome data with this small pilot study illustrate the potential for the use of DMO in non-psychiatric conditions such as hypertension and diabetes and may guide future research/incorporation of DMSs into medications for chronic illness where having the DMO data may allow for timely targeted interventions.

DMS use has been generally well-tolerated in studies [27,28,42]. Patients with an SMI generally experienced relatively minor dermatological adverse effects (ex: pruritus, rash) related to wearable sensor use [27,28,42]. In one SMI study, erythema led to the patient discontinuing participation in the trial [28]. In another study, one patient experienced a severe rash [42]. These results are consistent with studies in non-psychiatric conditions. In the Frias et al. study, rash from the sensor worn by the patient accounted for 13 out of 14 adverse events related to the device [22]. DiCarlo and colleagues reported that the most common adverse event related to the device was skin irritation to the sensor [37]. In the Eisenberger et al. study, six subjects stopped treatment early due to either “insufficient system usability” or rash. Rash was also reported by five other study subjects but did not lead to study discontinuation [36].

Some have expressed concern about DMS use in conditions with psychosis for possible worsening of paranoia due to “swallowing a spy”, in which the DMS reports out information about the patient [47]. Most patients are able to tell the difference “between a paranoid delusion and a voluntary contact with their doctor”, per John Kane, a psychiatrist who lead a feasibility study of aripiprazole with an ingestible sensor [47]. A study by Kane et al. excluded subjects with severe psychosis or symptoms of mania and did not find any of the subjects to experience paranoia of new-onset [27]. However, one patient with mild paranoia had exacerbation during the study, which was found not to be related to the study drug by the researchers [27]. Overall, in the studies, patients on oral aripiprazole were clinically stable and the majority had a clinical global impression severity of “mild disease” [27,28,42]. Given exclusion in trials, results may not be generalizable to unstable conditions with psychosis [27,28,42].

The usability of a DMS for the patient is a factor of consideration for optimal use [28,42,43]. Patients need to be able to use/engage with the technology and replace the patch [42]. Human factor studies allowed for the testing of DMSs in SMI to identify user-related risks and make modifications based on the findings [41]. Studies of DMSs in SMI show generally good usability [27,28,42]. The majority of the study completers thought a DHFS was easy to understand and could be useful for them [27]. Within eight weeks, the majority (82.1%) of subjects with schizophrenia in a phase IIa study by Peters-Strickland et al. were able to use it with little to no help [42]. However, patients with schizophrenia may need additional help when initiating a DMS compared with other conditions in SMI [28]. In the open-label pilot study evaluating a call center for a DMS in SMI, schizophrenia patients comprised half of the calls to the call center [28]. Per Kopelowicz et al., the greater average adherence rate in their study compared with the study by Peters-Strickland et al. may have been due to them focusing more on the “role of the integrated call center” [28]. Another consideration is that patients with severe symptoms were excluded from studies [27,28,42]. Therefore, the findings of these studies for usability in patients with a more mild and stable disease may not be generalizable to patients with an acute/more severe presentation [27,28,42].

## 5. Conclusions

An ingestible sensor may help address a gap in care by providing real-time objective medication-taking adherence information to clinicians. There is a lack of studies with information on treatment outcomes utilizing a DMS. Larger, controlled studies with active comparators measuring outcomes (ex: 30 day hospital readmission rate in SMI patients) are therefore needed to understand what role they can play in improving adherence. In addition, a limitation of the current literature is patients included in the SMI trials were generally controlled on their current oral antipsychotic and of mild severity. Therefore, we unable to generalize the results to a more severely ill SMI population. Future direction of clinical trials can help address some of these gaps to have a better understanding of the role of DMSs for improvement in adherence and on treatment outcomes.

## Figures and Tables

**Table 1:** Summary of Adherence with Ingestible Sensor Use.

Disease State	Authors	Patient Population	Study Design (Sample Size)	Medication Adherence (%) with Ingestible Sensor
**Cardiovascular**				
	Frias et al. [22]	Uncontrolled Hypertension and Type II Diabetes	Open-label cluster-randomized study (N = 109)	≥80%
	Dicarlo et al. [37]	Hypertension	Feasibility study (N = 37)	90%
**Psychiatry**				
	Kane et al. [27]	Bipolar Disorder and Schizophrenia	Observational study (N = 28)	74%
	Kopelowicz et al. [28]	Bipolar I Disorder, Major Depressive Disorder, Schizophrenia	Open-label, multicenter pilot study (N = 49)	88.6%
	Peters-Strickland et al. [41]	Schizophrenia	Multi-center, open-label phase IIa study (N = 67)	73.9%
**Transplant**				
	Eisenberger et al. [36]	Kidney Transplant	Open-label single-arm (N = 20)	99.4%

## References

[1] World Health Organization, World Health Organization (2003). Asthma. Adherence to Long-Term Therapies: Evidence for Action.

[2] Sendt K.V., Tracy D.K., Bhattacharyya S. (2015). A systematic review of factors influencing adherence to antipsychotic medication in schizophrenia-spectrum disorders. Psychiatry Res..

[3] Baumgartner P.C., Haynes R.B., Hersberger K.E., Arnet I. (2018). A Systematic Review of Medication Adherence Thresholds Dependent of Clinical Outcomes. Front. Pharmacol..

[4] Stentzel U., van den Berg N., Schulze L.N., Schwaneberg T., Radicke F., Langosch J.M., Freyberger H.J., Hoffmann W., Grabe H.J. (2018). Predictors of medication adherence among patients with severe psychiatric disorders: Findings from the baseline assessment of a randomized controlled trial (Tecla). BMC Psychiatry.

[5] Schulze L.N., Stentzel U., Leipert J., Schulte J., Langosch J., Freyberger H.J., Hoffmann W., Grabe H.J., van den Berg N. (2019). Improving Medication Adherence With Telemedicine for Adults With Severe Mental Illness. Psychiatr. Serv. (Wash. D.C.).

[6] Semahegn A., Torpey K., Manu A., Assefa N., Tesfaye G., Ankomah A. (2018). Psychotropic medication non-adherence and associated factors among adult patients with major psychiatric disorders: A protocol for a systematic review. Syst. Rev..

[7] Hurtado-de-Mendoza A., Cabling M.L., Sheppard V.B. (2015). Rethinking agency and medical adherence technology: Applying Actor Network Theory to the case study of Digital Pills. Nurs. Inq..

[8] Correll C.U., Lauriello J. (2018). Long-Acting Injectable Antipsychotics: Where Do They Fit in the Treatment Plan?. J. Clin. Psychiatry (Memphis T.N. USA).

[9] Kikkert M.J., Dekker J. (2017). Medication Adherence Decisions in Patients with Schizophrenia. Prim. Care Companion CNS Disord..

[10] Czobor P., Van Dorn R.A., Citrome L., Kahn R.S., Fleischhacker W.W., Volavka J. (2015). Treatment adherence in schizophrenia: A patient-level meta-analysis of combined CATIE and EUFEST studies. Eur. Neuropsychopharmacol..

[11] Greene M., Burudpakdee C., Seetasith A., Behling M., Krasa H. (2019). Evaluation of patient support program and adherence to long-acting injectable aripiprazole for patients utilizing injection local care centers. Curr. Med. Res. Opin..

[12] Limandri B.J. (2019). Long-Acting Injectable Antipsychotic Medications: Why Aren’t They Used as Often as Oral Formulations?. J. Psychosoc. Nurs. Ment. Health Serv..

[13] Taylor D.M., Velaga S., Werneke U. (2018). Reducing the stigma of long acting injectable antipsychotics—Current concepts and future developments. Nord. J. Psychiatry.

[14] Lexicomp Database (2020). Perseris Drug Monograph.

[15] Greene M., Yan T., Chang E., Hartry A., Touya M., Broder M.S. (2018). Medication adherence and discontinuation of long-acting injectable versus oral antipsychotics in patients with schizophrenia or bipolar disorder. J. Med. Econ..

[16] Lexicomp Database (2019). RisperiDONE Drug Monograph.

[17] Lexicomp Database (2019). Invega Trinza Drug Monograph.

[18] Stip E., Vincent P.D., Sablier J., Guevremont C., Zhornitsky S., Tranulis C. (2013). A randomized controlled trial with a Canadian electronic pill dispenser used to measure and improve medication adherence in patients with schizophrenia. Front. Pharmacol..

[19] Steinkamp J.M., Goldblatt N., Borodovsky J.T., LaVertu A., Kronish I.M., Marsch L.A., Schuman-Olivier Z. (2019). Technological Interventions for Medication Adherence in Adult Mental Health and Substance Use Disorders: A Systematic Review. JMIR Ment. Health.

[20] Haddad P.M., Brain C., Scott J. (2014). Nonadherence with antipsychotic medication in schizophrenia: Challenges and management strategies. Patient Relat. Outcome Meas..

[21] Kalantarian H., Motamed B., Alshurafa N., Sarrafzadeh M. (2016). A wearable sensor system for medication adherence prediction. Artif. Intell. Med..

[22] Frias J., Virdi N., Raja P., Kim Y., Savage G., Osterberg L. (2017). Effectiveness of Digital Medicines to Improve Clinical Outcomes in Patients with Uncontrolled Hypertension and Type 2 Diabetes: Prospective, Open-Label, Cluster-Randomized Pilot Clinical Trial. J. Med. Internet Res..

[23] Lexicomp Database (2019). Aripiprazole Drug Monograph.

[24] Otsuka America Pharmacetical (2017). Abilify Mycite Package Insert.

[25] Kane J.M. (2018). Comments on Abilify MyCite. Clin. Schizophr. Relat. Psychoses.

[26] (2019). Aripiprazole with digital ingestion tracking (Abilify MyCite). Med. Lett. Drugs Ther..

[27] Kane J.M., Perlis R.H., DiCarlo L.A., Au-Yeung K., Duong J., Petrides G. (2013). First experience with a wireless system incorporating physiologic assessments and direct confirmation of digital tablet ingestions in ambulatory patients with schizophrenia or bipolar disorder. J. Clin. Psychiatry (Memphis T.N. USA).

[28] Kopelowicz A., Baker R.A., Zhao C., Brewer C., Lawson E., Peters-Strickland T. (2017). A multicenter, open-label, pilot study evaluating the functionality of an integrated call center for a digital medicine system to optimize monitoring of adherence to oral aripiprazole in adult patients with serious mental illness. Neuropsychiatr. Dis. Treat..

[29] Flores G.P., Peace B., Carnes T.C., Baumgartner S.L., Buffkin D.E., Euliano N.R., Smith L.N. (2016). Performance, Reliability, Usability, and Safety of the ID-Cap System for Ingestion Event Monitoring in Healthy Volunteers: A Pilot Study. Innov. Clin. Neurosci..

[30] Mukete B.N., Ferdinand K.C. (2016). Polypharmacy in Older Adults with Hypertension: A Comprehensive Review. J. Clin. Hypertens. (Greenwich C.T. USA).

[31] Holender A., Sutton S., De Simoni A. (2018). Opinions on the use of technology to improve tablet taking in >65-year-old patients on cardiovascular medications. J. Int. Med. Res..

[32] Garrison L.E., Haberer J.E. (2017). Technological methods to measure adherence to antiretroviral therapy and preexposure prophylaxis. Curr. Opin. HIV AIDS.

[33] Subbaraman R., de Mondesert L., Musiimenta A., Pai M., Mayer K.H., Thomas B.E., Haberer J. (2018). Digital adherence technologies for the management of tuberculosis therapy: Mapping the landscape and research priorities. BMJ Glob. Health.

[34] Chai P.R., Carreiro S., Innes B.J., Chapman B., Schreiber K.L., Edwards R.R., Carrico A.W., Boyer E.W. (2017). Oxycodone Ingestion Patterns in Acute Fracture Pain With Digital Pills. Anesth. Analg. (Phila. P.A. USA).

[35] Chai P.R., Carreiro S., Innes B.J., Rosen R.K., O’Cleirigh C., Mayer K.H., Boyer E.W. (2017). Digital Pills to Measure Opioid Ingestion Patterns in Emergency Department Patients with Acute Fracture Pain: A Pilot Study. J. Med. Internet Res..

[36] Eisenberger U., Wuthrich R.P., Bock A., Ambuhl P., Steiger J., Intondi A., Kuranoff S., Maier T., Green D., DiCarlo L. (2013). Medication adherence assessment: High accuracy of the new Ingestible Sensor System in kidney transplants. Transplantation.

[37] DiCarlo L.A., Weinstein R.L., Morimoto C.B., Savage G.M., Moon G.L., Au-Yeung K., Kim Y.A. (2016). Patient-Centered Home Care Using Digital Medicine and Telemetric Data for Hypertension: Feasibility and Acceptability of Objective Ambulatory Assessment. J. Clin. Hypertens. (Greenwich C.T. USA).

[38] Noble K., Brown K., Medina M., Alvarez F., Young J., Leadley S., Kim Y., DiCarlo L. (2016). Medication adherence and activity patterns underlying uncontrolled hypertension: Assessment and recommendations by practicing pharmacists using digital health care. J. Am. Pharm. Assoc. JAPhA.

[39] Ibrahim M.E., Brooks K.M., Castillo-Mancilla J.R., McHugh C., Morrow M., Brothers J., MaWhinney S., Hosek S., Huhn G., Anderson P.L. (2018). Short Communication: Bioequivalence of Tenofovir and Emtricitabine After Coencapsulation with the Proteus Ingestible Sensor. AIDS Res. Hum. Retrovir..

[40] Rohatagi S., Profit D., Hatch A., Zhao C., Docherty J.P., Peters-Strickland T.S. (2016). Optimization of a Digital Medicine System in Psychiatry. J. Clin. Psychiatry (Memphis T.N. USA).

[41] Peters-Strickland T., Hatch A., Smith J., Bartfield B., Pestreich L., Rohatagi S., Forma F., Raja P., Docherty J. Human Factors Evaluation of a Novel Digital Medicine System in Psychiatry. Proceedings of the International Society for CNS Clinical Trials and Methodology 12th Annual Meeting.

[42] Peters-Strickland T., Pestreich L., Hatch A., Rohatagi S., Baker R.A., Docherty J.P., Markovtsova L., Raja P., Weiden P.J., Walling D.P. (2016). Usability of a novel digital medicine system in adults with schizophrenia treated with sensor-embedded tablets of aripiprazole. Neuropsychiatr. Dis. Treat..

[43] Hatch A., Docherty J.P., Carpenter D., Ross R., Weiden P.J. (2017). Expert Consensus Survey on Medication Adherence in Psychiatric Patients and Use of a Digital Medicine System. J. Clin. Psychiatry (Memphis T.N. USA).

[44] Profit D., Rohatagi S., Zhao C., Hatch A., Docherty J.P., Peters-Strickland T.S. (2016). Developing a Digital Medicine System in Psychiatry: Ingestion Detection Rate and Latency Period. J. Clin. Psychiatry (Memphis T.N. USA).

[45] Chai P.R., Castillo-Mancilla J., Buffkin E., Darling C., Rosen R.K., Horvath K.J., Boudreaux E.D., Robbins G.K., Hibberd P.L., Boyer E.W. (2015). Utilizing an Ingestible Biosensor to Assess Real-Time Medication Adherence. J. Med. Toxicol..

[46] Cosgrove L., Cristea I.A., Shaughnessy A.F., Mintzes B., Naudet F. (2019). Digital aripiprazole or digital evergreening? A systematic review of the evidence and its dissemination in the scientific literature and in the media. BMJ Evid. Based Med..

[47] Rosenbaum L. (2018). Swallowing a Spy—The Potential Uses of Digital Adherence Monitoring. N. Engl. J. Med..

